# Comparative analysis of the sensitivity of metagenomic sequencing and PCR to detect a biowarfare simulant (*Bacillus atrophaeus*) in soil samples

**DOI:** 10.1371/journal.pone.0177112

**Published:** 2017-05-04

**Authors:** Delphine Plaire, Simon Puaud, Marie-Claude Marsolier-Kergoat, Jean-Marc Elalouf

**Affiliations:** 1 CNRS-UMR 7206, Eco-anthropologie et Ethnobiologie, Département Hommes, Natures et Sociétés, Musée de l’Homme, 17 place du Trocadéro et du 11 novembre, Paris, France; 2 Institute for Integrative Biology of the Cell (I2BC), IBITECS, CEA, CNRS, Université Paris‐Sud, Université Paris‐Saclay, Gif‐sur‐Yvette cedex, France; 3 CNRS-UMR 7194, Histoire naturelle de l’Homme préhistorique, Département Préhistoire MNHN—UPVD—Sorbonne Université- Musée de l’Homme, 17 Place du Trocadéro et du 11 novembre, Paris, France; University of Helsinki, FINLAND

## Abstract

To evaluate the sensitivity of high-throughput DNA sequencing for monitoring biowarfare agents in the environment, we analysed soil samples inoculated with different amounts of *Bacillus atrophaeus*, a surrogate organism for *Bacillus anthracis*. The soil samples considered were a poorly carbonated soil of the silty sand class, and a highly carbonated soil of the silt class. Control soil samples and soil samples inoculated with 10, 10^3^, or 10^5^ cfu were processed for DNA extraction. About 1% of the DNA extracts was analysed through the sequencing of more than 10^8^ reads. Similar amounts of extracts were also studied for *Bacillus atrophaeus* DNA content by real-time PCR. We demonstrate that, for both soils, high-throughput sequencing is at least equally sensitive than real-time PCR to detect *Bacillus atrophaeus* DNA. We conclude that metagenomics allows the detection of less than 10 ppm of DNA from a biowarfare simulant in complex environmental samples.

## Introduction

The 2001 bioterrorism incident in the USA led France to develop in 2003 Biotox-Piratox laboratories to detect biological and chemical risks caused by pathogen agents. The organism used for this attack was *Bacillus anthracis*, the causative agent of anthrax [1]. In 2005, the French government launched a program against chemical, biological, radiological, and nuclear threats (CBRN).

In the years following the anthrax threat, there was an increasing demand for developing methods that allow the detection of bioterrorism agents. The key points that need to be addressed by these methods are the specific, sensitive and rapid detection of pathogen agents [2]. Before 2001, only few studies had been performed in the field of fast pathogen detection. Interestingly, one of them delivered a PCR assay that enabled the detection of bacterial DNA in a few minutes [3]. After the 2001 events in the USA, a lot of researches were launched to elaborate fast and easy methods to detect pathogen agents. Several types of methods are used to detect and identify biothreat agents.

Biochemical characteristics have been considered to identify biothreat agents. Substrate utilization patterns as well as fatty acid profiling have been used to detect and differentiate *Yersinia spp*. (reviewed in [2]). Immunological tests have been improved for the detection of infectious diseases, drugs, toxins and pollutants [4]. A number of assays are similar to the classic sandwich assay based on the enzyme-linked immunosorbent assay (ELISA). Laporte et al. [5] developed dipstick assays to detect the pathogens *Yersinia enterocolitica* and *Yersinia pseudotuberculosis*. This method is simple, fast, inexpensive, and can be used directly by non-specialists in the field. However, the specificity of immunoassays is limited by antibody quality and their sensitivity is usually lower than that of PCR and other assays based on DNA amplification. Immunological methods can be combined with other approaches such as mass spectrometry to analyse bacterial proteins in complex matrices. Martelet et al. [6] demonstrated that an assay resting on phage amplification, immunomagnetic separation and mass spectrometry allows to detect *E*. *coli* cells in food samples with a sensitivity in the range of 1 colony-forming unit (cfu)/ml, corresponding to 10 cfu/assay. Mass spectrometry can also be used as a standalone method for the analysis of pathogenic bacteria [7].

Many genetic studies were initiated with the goal of providing methods for fast detection of pathogens. Real-time PCR is well suited for this purpose since it combines PCR sensitivity with the simultaneous detection of the target. Amplification monitoring can be performed using the intercalating dye SYBR Green or fluorogenic probes (such as TaqMan probes), the later detection system being much more specific than the former. Real-time PCR assays, including assays compatible with handheld devices [8], are available for a number of biothreat agents such as ricin [9], *Bacillus anthracis* [10–11], *Yersinia pestis* [11], and emetic *Bacillus cereus* [12].

Since 2005, next-generation sequencing (NGS) methods have been developed and currently produce millions of sequences in a short time and at low cost. These new technologies boosted metagenomic studies, *i*.*e*. the analysis of the full DNA material of complex samples. In a bioterrorism context, metagenomic sequencing allows the analysis of any pathogenic organism whose genomic sequence is available, including genetically modified versions of pathogens and unculturable microorganisms [13].

In order to avoid the manipulation of pathogenic agents, surrogate organisms, which are non-pathogenic, are commonly used. In *Bacillus anthracis* studies, it is common to use *Bacillus thuringiensis* [14] or *Bacillus atrophaeus* [15–18] as surrogates. In our study, *Bacillus atrophaeus*, a gram-positive bacterium, was used.

The aim of this study was to compare the performance of metagenomic DNA sequencing with TaqMan-based real-time PCR for analysing environmental samples. Studies by Be et al. [19] demonstrated that metagenomic sequencing allows to detect 10–100 genomic equivalents (GEs) of *Bacillus anthracis* DNA added to aerosol or soil DNA extract. Here, we directly added bacterial cells to environmental samples, and extracted simultaneously the contaminant and the endogenous DNA of the sample. A rapid DNA extraction procedure was used, and techniques were adapted to reach high sensitivity by analysing a large number of DNA fragments.

## Materials and methods

### Collection, characterization and texture analysis of soil samples

Two soil samples were collected from different areas in France: soils A and B come from Saclay (Essone), and Reims (Marne), respectively. No specific permissions were required for soil sample acquisition in the field, as all collections were performed on public, non-protected land. These field studies did not involve any endangered or protected species. For the determination of soil colour we used the Munsell soil-colour chart [20]. The calcium carbonate (CaCO_3_) content was measured with a Bernard’s calcimeter [21], and grain-size distributions (raw and decarbonated sediment) were characterized on the fine fraction (after sieving at 2 mm) using a Malvern Mastersizer 2000 (Malvern, UK) laser granulometer. For soil class determination, data were plotted in a Groupe d’Étude pour les Problèmes de Pédologie Appliquée (GEPPA) soil texture triangle diagram [22].

### DNA extraction from soil samples

The *Bacillus atrophaeus* strain 930029 [23] was cultured in lysogeny broth medium (LB) to a 605 nm OD of 0.4. The number of living cells, evaluated by plating serial dilutions of the culture on Petri dishes, corresponded to 10^8^ cfu/ml. Aliquots (750 μl of the bacterial cell culture supplemented with 250 μl of glycerol 80%) were stored at -80°C. Soil samples (250 mg) were inoculated with serial dilutions of the same bacterial frozen aliquot corresponding to absolute amounts of 10, 10^3^, or 10^5^ cfu. No bacteria were inoculated in control samples. DNA was extracted from soils with the PowerSoil DNA Isolation Kit (Mobio, Carlsbad, CA) using procedures recommended by the manufacturer. DNA was recovered as a 100-μl sample volume. Analysis of DNA in the extracts using a NanoDrop 2000 spectrophotometer (Thermo Fisher Scientific) yielded values below those that can be measured accurately (<0.1 OD_260nm_, *i*.*e*. <5 ng/μl).

### PCR analysis

For PCR-amplification of *Bacillus atrophaeus* DNA, we first designed primers encompassing a 107-bp fragment of a gene predicted to encode a sodium:solute symporter, corresponding to nucleotides 46,317–46,423 of the reference genome (GenBank accession number NC_014639) derived from the studies by Gibbons et al. [15]. The forward (5’-GCCACATATAGAAACCCAACAC-3’) and reverse (5’-AGTACATACAGCGAACCCT-3’) primers were designed with the help of Oligo 7 software (Cascade, CO, USA). The specific amplification of *Bacillus atrophaeus* DNA from our samples was checked by analysing DNA extracts of control soil samples and of soil samples inoculated with *Bacillus atrophaeus*. Amplification was carried out in a 50-μl reaction volume containing 300 nM of sense and antisense primers, 200 μM dNTP, 2.5 mM MgCl_2_, 5 μl of GeneAmp10X PCR buffer II, 2.5 U of AmpliTaq Gold DNA polymerase (Thermo Fisher Scientific, Waltham, MA, USA), and water (blank samples) or serial dilutions of the DNA extracts, from 7.3 μl to 0.01 μl. After an activation step (95°C, 8.5 min), 45 PCR cycles (95°C, 15 s; 56°C, 20 s; 70°C, 1 min) were performed in a Veriti thermal cycler (Thermo Fisher Scientific). PCR samples were electrophoresed through an 8% polyacrylamide gel, then the gel was stained with SYBR Green I (Thermo Fisher Scientific) and illuminated with UV light for DNA visualisation. No amplification products were obtained from control soil samples, whereas a single band of the expected size was consistently detected in soil samples inoculated with *Bacillus atrophaeus*, indicating that the 107-bp fragment of the *Bacillus atrophaeus* genome is a relevant target for PCR analysis. These experiments also demonstrated that the PCR DNA yield decreased when the amount of extract was higher than 2.4 μl. In line with previous interpretation for experiments carried out on environmental samples [24–27], we concluded that the DNA extracts contained PCR inhibitors and we used limited amounts of the DNA extracts in subsequent experiments.

To set up a real-time PCR assay, we analysed the 107-bp DNA fragment with the custom TaqMan assay design tool (Thermo Fisher Scientific). The sequences of the PCR primers and the TaqMan-MGB probe are as follows: forward primer, 5’- ACAGTGGAGATTAGCCAAGAAACAC-3’; reverse primer, 5’- GTACATACAGCGAACCCTGAATTTC-3’; and probe, 5’FAM- CTGGTCAGGGAACTTC-NFQ-MGB3’. Real-time PCR was carried out in a 20-μl reaction volume containing 10 μl of 2X TaqMan universal PCR Master Mix, 1 μl of the probe-primers mixture, and water or DNA extracts. Amplification was performed in a CFX96 touch real-time PCR detection system (BioRad, Hercules, CA, USA) and included an initial step for enzyme activation (95°C, 10 min) followed by 40 PCR cycles (95°C, 15 s; 60°C, 1 min). Data were analysed by calculating the cycle threshold (*C*_T_), which corresponds to the number of PCR cycles required for the fluorescent signal to exceed the background level. For calibration purposes, known amounts of *Bacillus atrophaeus* genomic DNA were analysed with our TaqMan assay. The template used in these experiments consisted in *Bacillus atrophaeus* genomic DNA extracted from an exponentially growing culture of *Bacillus atrophaeus* cells. The concentration of the DNA stock solution (40 ng/μl), was measured using a NanoDrop 2000 spectrophotometer. The amplification efficiency of the TaqMan assay was calculated using the equation E = (10^−1/slope^ -1) x 100.

### Metagenomic analyses

#### DNA shearing

To shear DNA into 150-bp fragments, the soil DNA extracts were sonicated using the ultrasonicator Covaris S220 with microtube-15 (Covaris, MA, USA). The protocol provided by the manufacturer was used to perform the fragmentation following the parameters indicated for a 150 bp cut-off (peak incident power, 18 W; duty factor 20%; cycles per bust, 50; duration, 300 s).

#### Generation and sequencing of libraries of DNA fragments

Libraries of DNA fragments were generated for Illumina sequencing [28] using the Illumina TruSeq Nano DNA LT sample kit (San Diego, CA, USA). Eight libraries were produced, corresponding to 4 experimental conditions (0, 10, 10^3^, and 10^5^ cfu inoculated) for each 250 mg soil sample. The process of library construction, adapted from the Illumina TruSeq procedures for the purpose of analysing short (~ 150-bp) DNA fragments, consisted of 5 steps. First, 5 μl of sheared DNA (i.e. 5% of each DNA extract) was 5’end-phosphorylated and blunt-ended in a 100-μl reaction volume containing T4 polynucleotide kinase, T4 and Klenow DNA polymerases, and the reaction product was purified on a MinElute column (Qiagen, Hilden, Germany). The amount of DNA extract used to produce the libraries was selected based on previous experiments demonstrating that the extracts contained PCR inhibitors (see above). Second, a 3’ adenine residue was added to the blunt-ended DNA fragments using Klenow 3’ to 5’ exo- polymerase, and the enzyme was heat-inactivated at 70°C. Third, Illumina adapters with an overhanging thymine were ligated to the DNA fragments; the reaction product was purified using a Qiaquick PCR purification column (Qiagen) and recovered in a volume of 30 μl referred to as the DNA library. Fourth, a 5-μl aliquot of the library (corresponding to 0.83% of the DNA extract) was PCR-amplified using Phusion DNA polymerase (95°C, 3 min for enzyme activation, followed by 12 PCR cycles of 98°C for 20s, 60°C for 15s, and 72°C for 30s). Fifth, the full PCR reaction volume was loaded on an 8% polyacrylamide gel stained with SYBR Green I, and the 150–300 bp long DNA fragments (consisting of 122 bp derived from the adapters, 30 to 180 bp derived from the sample) were cut off the gel, purified and recovered in a final volume of 30 μl.

DNA sequencing was performed at Genoscope (Evry, France). DNA concentration in the amplified libraries was measured by quantitative PCR using Illumina primers and ranged between 1.5 and 4.0 ng/μl. Sequencing was carried out on the Illumina HiSeq 2500 platform using HiSEQ v3 chemistry with a read length set to 101 nucleotides and analysis on the single read mode. The eight libraries of the present project were sequenced on an 8-lane flow-cell, with a single library loaded on each lane. The DNA reads of this study have been deposited at EBI under accession numbers ERX1944760-ERX1944767.

#### Sequences analyses

Reads were trimmed for adapter sequences, N’s and low quality stretches on the 3’ end, using a software based on the FASTX-Toolkit package (http://hannonlab.cshl.edu/fastx_toolkit) and designed by Genoscope. The Illumina reads, ranging in size from 30 to 101 nucleotides, were aligned to a set of 15 bacterial genomes, including the reference genome of *Bacillus atropheus* (GenBank accession number NC_014639.1) and the genomes of bacterial species more or less closely related to *Bacillus atropheus* that are of interest to assess the specificity of the alignment analysis [10, 19, 29–30]. These include *Bacillus anthracis* strain 'Ames Ancestor' (NC_007530.2), *Bacillus anthracis* strain Sterne (NC_005945.1), *Bacillus cereus* (NC_014335.1), *Bacillus thuringiensis* (NC_008600.1), *Bacillus mycoides* (NZ_CP009692.1), *Bacillus megaterium* (NC_014019.1), *Bacillus subtilis* (NC_000964.3), *Geobacillus stearothermophilus* (NZ_CM002692.1), *Bacillus amyloliquefaciens (NZ_CP011278.1), Bacillus licheniformis* (NZ_CP014781.1), *Bacillus pseudomycoides* (NZ_CM000745.1), *Bacillus pumilus* (NZ_CP014165.1), *Lysinibacillus sphaericus* (NZ_CP015224.1) and *Bacillus weihenstephanensis* (NZ_CP009746.1). The alignments were carried out using BWA version 0.5.7 [31] with default options except for -*o* (gap opening) and -*n* (maximum edit distance), which were both set to 0, thus requiring a perfect match between the reads and the reference sequence. To evaluate the number of reads matching each bacterial genome, the reads were aligned to each genome independently and only the reads with a minimal mapping quality of 25 were taken into account. The reads matching specifically the *Bacillus atropheus* genome were extracted by aligning the reads to the 15 bacterial genomes simultaneously and retaining the reads mapping exclusively to the *Bacillus atropheus* genome with a minimal mapping quality of 25. Duplicate reads were discarded in all cases.

The species composition of each control soil (no inoculated cells) was examined by analysing one million Illumina reads using BLAST+ [32] against the GenBank *nt/nr* database with the following options: -task megablast -word_size 19 -max_target_seqs 1 -gapopen 5 -gapextend 2. Only the hits that display an E-value lower than 0.01 were considered significant.

## Results

**Fig 1:**
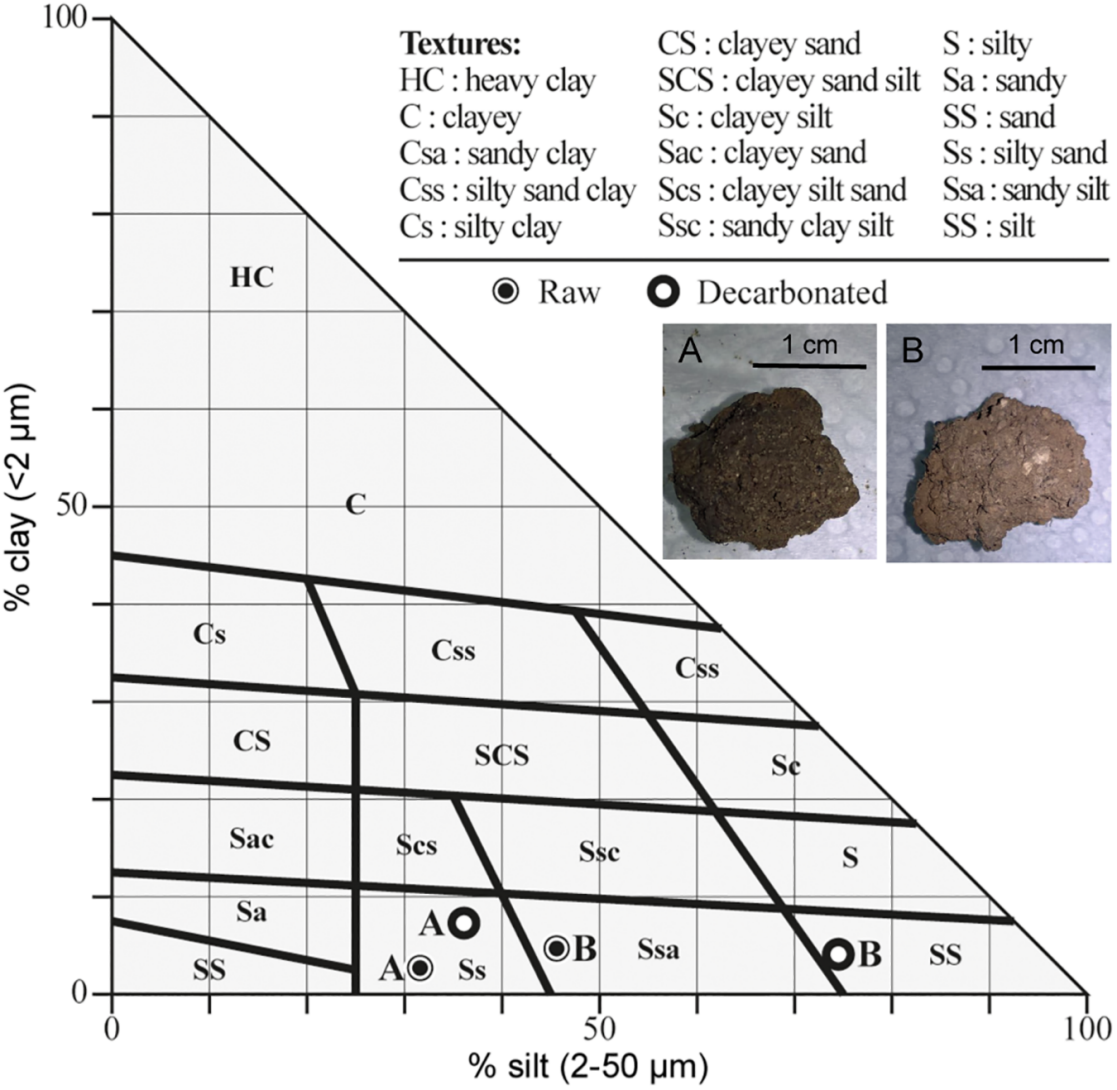
GEPPA soil texture triangle diagram. Grain size distribution data are plotted for raw and decarbonated soil A and soil B samples. Inserts show pictures of soils A and B.

We analysed two soil samples originating from Saclay (Essone, France, soil A) and Reims (Marne, France, soil B). Soil A displays a dark, grey brown colour, indicative of a high organic content. The raw fraction of soil A has a low coarse sand content (Table 1). Soil B colour is light grey, with a high content of coarse sand (granules of limestone sieving at 2 mm) in the raw fraction. Overall, the raw material of both soils displays a texture where silt and sand (fine plus coarse) predominate. Table 1 also shows that after CaCO_3_ dissolution with hydrochloric acid, the grain size distribution of soil A does not change dramatically, whereas silt material turns out to be predominant in soil B. Plotting the data in a GEPPA soil texture triangle diagram (Fig 1) shows that soil A contains no major coarse fraction, is poorly carbonated, and positions in the silty sand class (Ss). Raw soil B is plotted in the sandy silt class (Ssa) and, when decarbonated, the sample positions in the silt class (SS). We conclude from these analyses that soil A developed from Quaternary aeolian silt (*i*.*e*. loess), and likely corresponds to a decarbonated surface horizon (CaCO_3_ content: 4%). It is more humic than soil B, which is highly carbonated (CaCO_3_ content: 70%), and developed on a limestone (chalk) substratum, with rock fragments integrated in the soil.

**Table 1:** Grain size distribution and parameters for soil A and soil B.

Sample		Grain size distribution (%)				Grain size parameters			Sorting Index ^a^
		Clay	Silt	Fine sand	Coarse sand	Mode(s) (μm)	Mean (μm)	Median (μm)	
Raw	A	4.7	45.6	37.7	12.0	32 / 150	84	49	2.6
	B	7.3	36.1	26.7	30.0	89 / 677	232	70	4.6
Decarbonated	A	2.7	31.6	51.2	14.5	146	103	90	2.4
	B	4.1	74.5	15.6	5.8	22	51	18	2.4

Grain size distribution and grain size parameters: clay, 0.02 to 2 μm; silt, 2 to 50 μm; fine sand, 50 to 200 μm; coarse sand, 200 μm to 2 mm.

^a^ Trask [33] sorting index.

**Fig 2:**
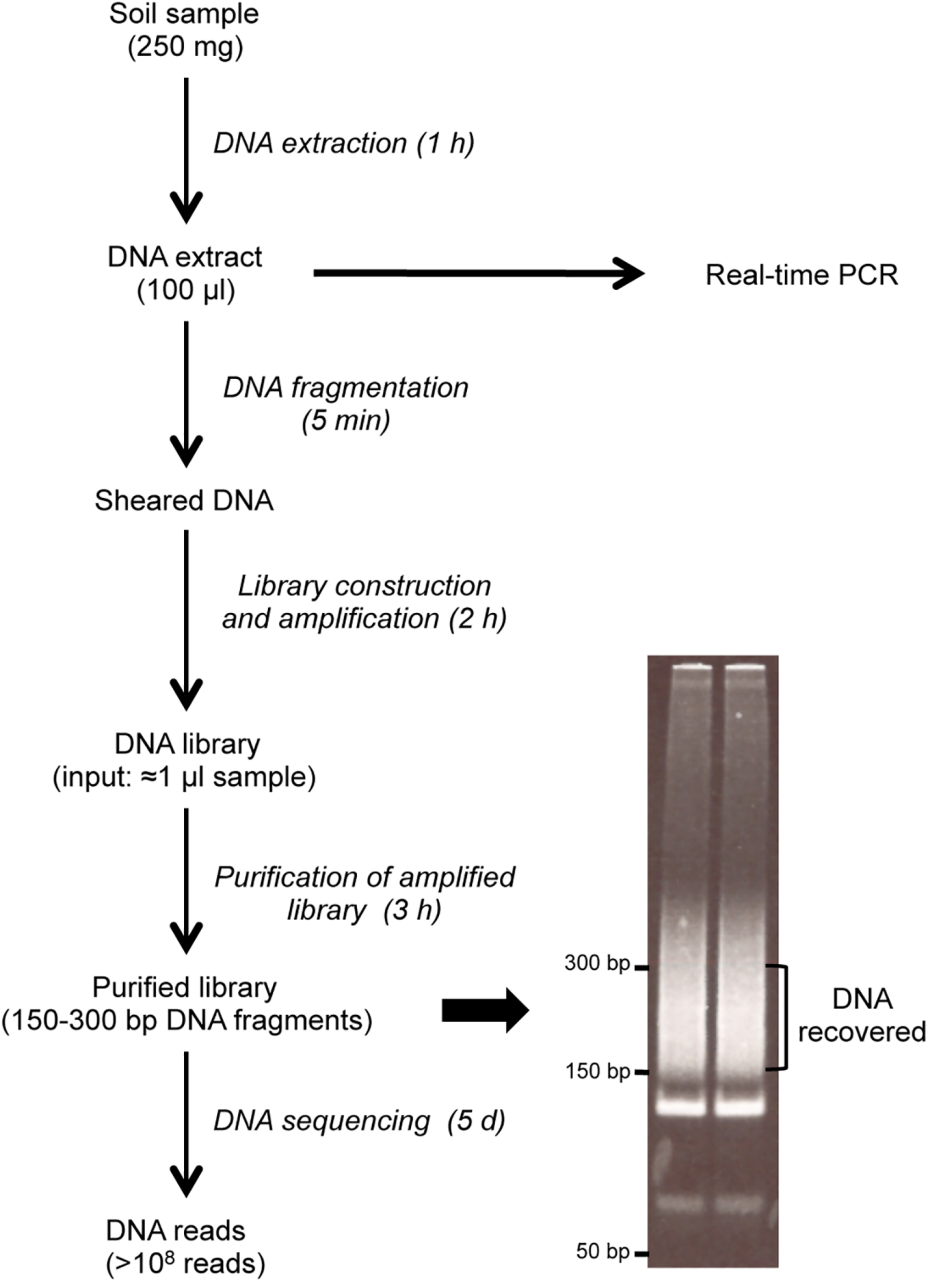
Schematic of the experimental workflow for metagenomic and PCR analysis of soil samples.

Fig 2 displays the workflow of our analytical pipeline for molecular studies. A 250 mg soil sample (either a control soil or soil sample inoculated with 10−10^5^ *Bacillus atrophaeus* cfu) is processed to recover DNA in a volume of 100 μl. For PCR studies, we analysed aliquots of the DNA extract. For high-throughput sequencing, a library has to be produced from short DNA fragments. We therefore sheared DNA to obtain DNA fragments in the 150-bp range, and produced the amplified library from DNA amounts corresponding to 0.83 μl (*i*.*e*. 0.83%) of the extract. Fragments of the amplified library ranging in size between 150 and 300-bp (122-bp of adapter sequences, and 30 to 180-bp soil DNA fragments) were then purified on a polyacrylamide gel and shotgun-sequenced on the Illumina Hiseq 2500 platform on the normal mode, yielding more than 10^8^ reads for each library. The process of DNA extraction and library construction can be performed within one working day. DNA sequencing on the Hiseq 2500 platform required 5 days using the normal mode.

**Fig 3:**
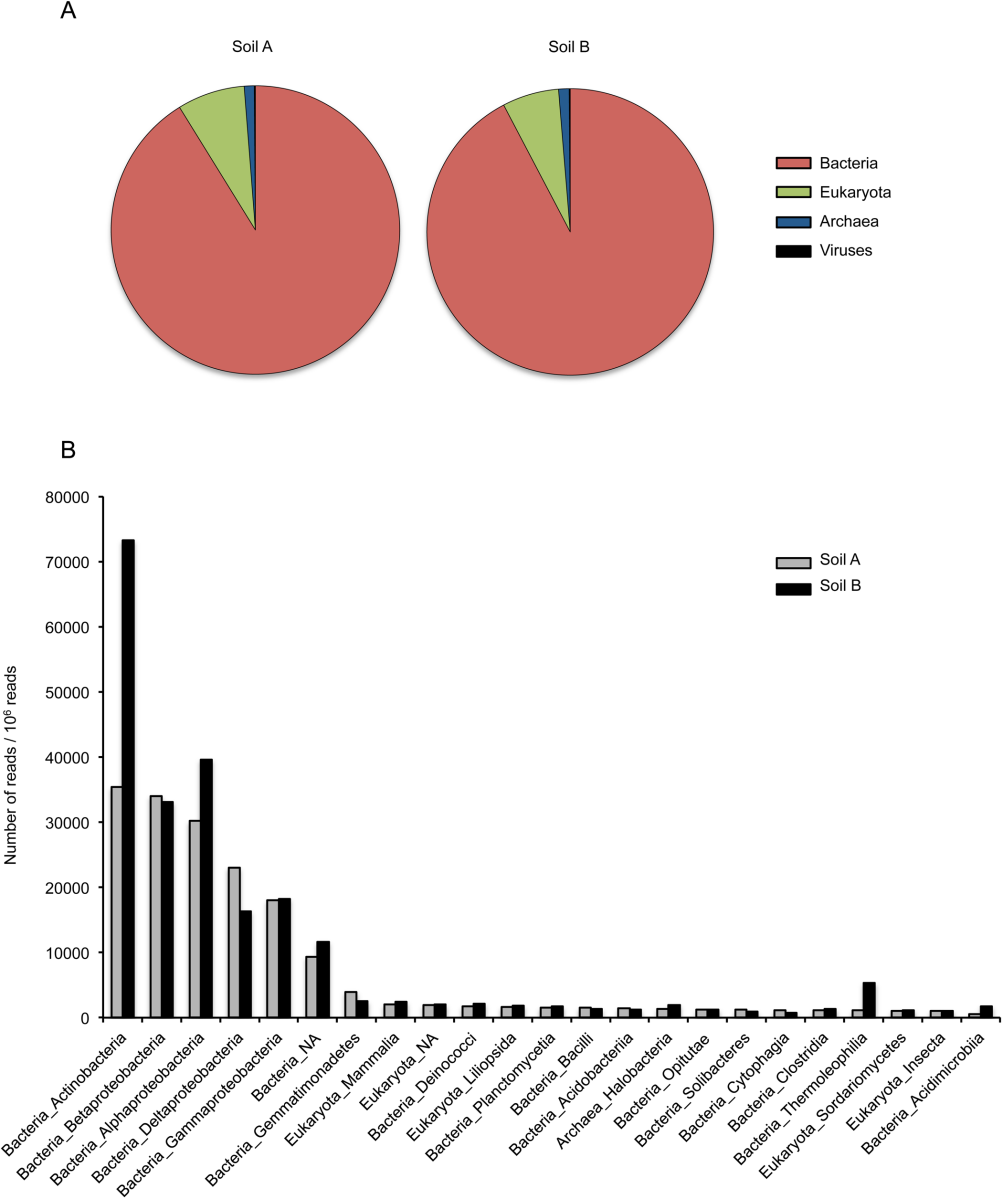
Metagenome data for control soils. (A) Proportion of DNA for Bacteria, Eukaryota, Archaea, and viruses detected in a random subset of 10^6^ reads from each soil sample. Only DNA reads that display an E-value lower than 0.01 for BLAST alignment to the *nt/nr* database were considered. This corresponds to 19.0 and 23.7% of all reads for soils A and B, respectively. In soil A, Bacteria, Eukaryota, Archaea, and viruses correspond to 91.2%, 7.6%, 1.1% and 0.1% of assigned reads. In soil B, Bacteria, Eukaryota, Archaea, and viruses correspond to 92.3%, 6.4%, 1.2% and 0.1% of assigned reads. (B) Organism classes detected through a minimum of 1,000 DNA reads in at least one soil sample.

To get insight into the metagenome of the two soil samples, each control library (*i*.*e*. samples not inoculated with *Bacillus atrophaeus* cells) was analysed for a random subset of 10^6^ reads that were aligned using BLAST+ to the *nt/nr* database. As usually observed with environmental samples [34–35] only a minor fraction of the DNA reads (soil A: 19.0%; soil B: 23.7%) could be assigned a taxonomic identification. Fig 3A shows that for the two soil samples the mapped reads mostly corresponded to bacterial DNA, followed in decreasing order by Eukaryota, Archaea, and virus DNA. We also analysed the two soil samples for the organism classes that were detected through more than 0.1% of the reads (10^3^ out of the 10^6^ reads analysed by BLAST+). As shown in Fig 3B, the same classes ranked first in both samples. Actinobacteria, Alphaproteobacteria, Betaproteobacteria, Deltaproteobacteria, and Gammaproteobacteria were especially abundant, in agreement with the previously reported structure of a soil metagenome [36]. However, a two- to five-fold difference was observed between the two soils for the abundance of three Bacteria classes (Actinobacteria, Thermoleophilia, and Acidimicrobiia).

**Fig 4:**
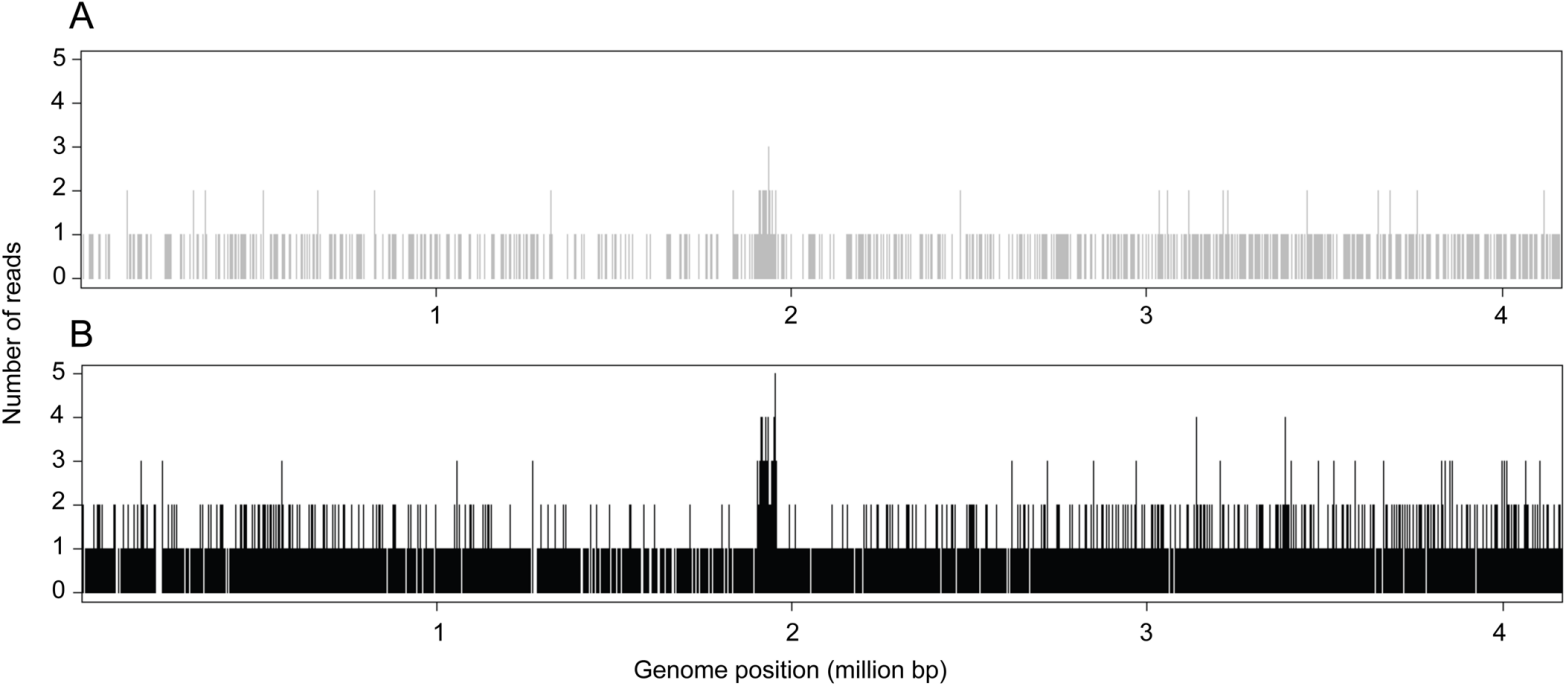
*Bacillus atrophaeus* genome coverage. The figure shows the number of DNA reads overlapping each position of the *Bacillus atrophaeus* genome. (A) Soil A sample inoculated with 10^5^ cfu; (B) Soil B sample inoculated with 10^5^ cfu.

We next attempted to identify *Bacillus atrophaeus* DNA in all 8 libraries of our metagenome dataset. For this, the sequencing reads were aligned on a set of 15 genomes, including the *Bacillus atrophaeus* reference genome and bacterial genomes that were used to assess the specificity of the mapping analysis (see Materials and Methods). S1 Table shows the number of reads that mapped to each genome, including the reads that mapped to several genomes. This analysis yielded an insight into the variability of the number of DNA reads mapping to a variety of *Bacillus* sp. genomes from one library to another. Important variations were noted for a few species (e.g. *Bacillus mycoides* and *Bacillus weihenstephanensis* in soil B). With regard to the DNA reads aligned to the *Bacillus atrophaeus* genome, a more than 100-fold difference as compared to control soil samples, and a high number of reads were only obtained in soil samples inoculated with 10^5^ cfu. Table 2 shows that most of these reads (750 out of 754 for soil A, and 3,122 out of 3,128 for soil B) aligned to the *Bacillus atrophaeus* genome but to none of the other 14 bacilli genomes, indicating that a specific signal was recorded. The larger number of *Bacillus atrophaeus* reads in soil B than in soil A could indicate a larger proportion of *Bacillus atrophaeus* DNA as compared to the DNA from endogenous organisms in one soil than in the other. As expected for a shotgun sequencing approach, the location of DNA reads was almost evenly distributed throughout the *Bacillus atrophaeus* genome, as shown in Fig 4. A higher density of reads was recorded between nucleotide 1,952,509 and 1,952,745. In the Genbank report, this specific region is referred to as a CDS for a hypothetical protein, with the annotation derived by automated computational analysis using gene prediction method. BLAST analysis of this CDS against the *nt/nr* database only yielded significant alignment parameters with *Bacillus atrophaeus* strains (E value = 2x10^-116^) and no significant alignment with any other organisms (E value > 0.076).

**Table 2:** Results of DNA sequence analyses.

	Total number of reads		Reads aligned to *Bacillus atrophaeus* genome			
			Number of reads		Number of reads/10^8^ reads	
Inoculated cfu	Soil A	Soil B	Soil A	Soil B	Soil A	Soil B
0	113,898,947	142,037,275	1	0	1	0
10	127,912,763	129,309,250	1	0	1	0
10^3^	129,291,436	192,572,216	13	77	10	40
10^5^	125,261,786	148,385,427	939	4633	750	3122

For each library (produced from 0.83% of the total DNA extract), the table shows the total number of DNA reads and the number of unique reads that align to the *Bacillus atrophaeus* genome but not to any of the other genomes listed in S1 Table.

**Fig 5:**
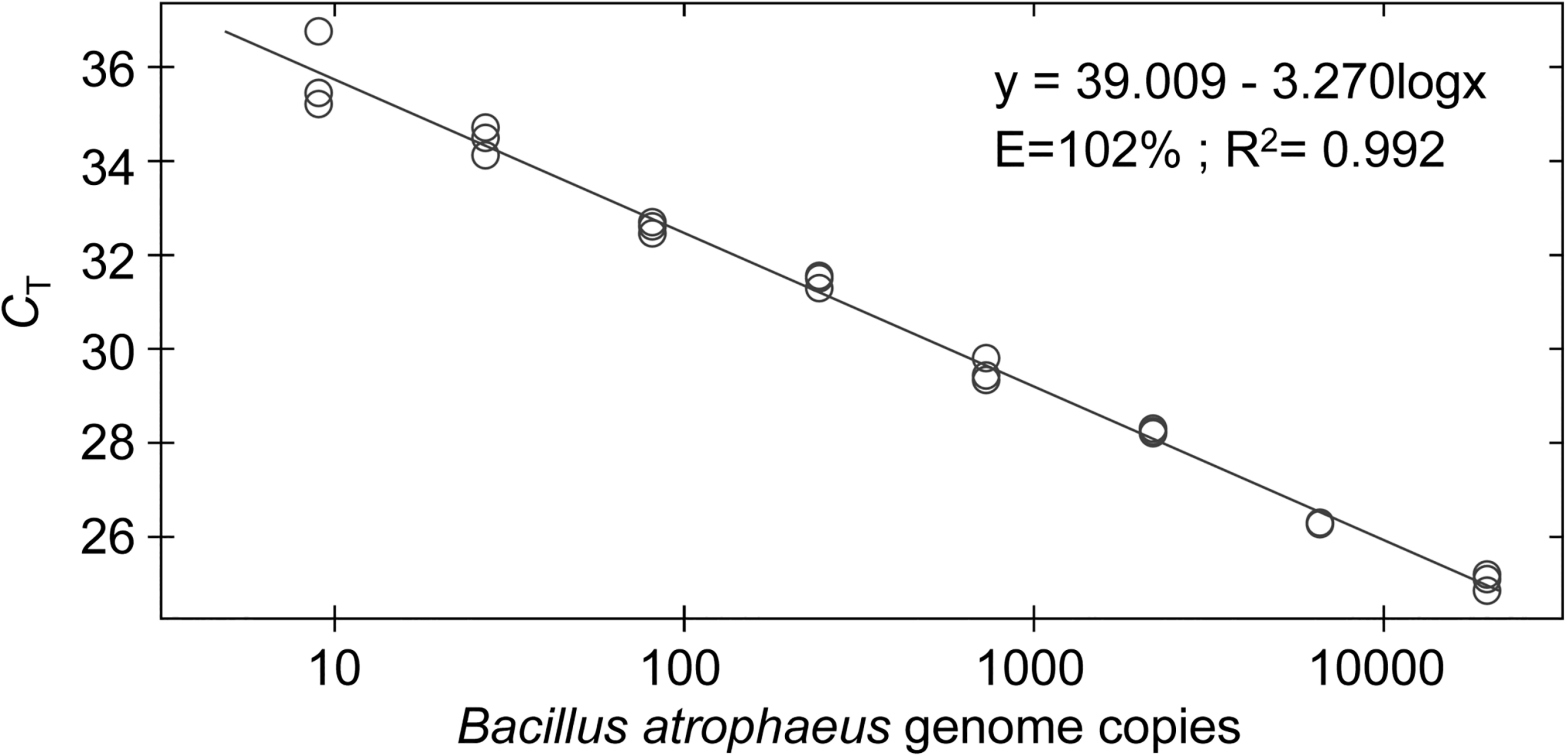
Sensitivity of the TaqMan real-time PCR assay. Serial dilutions of a *Bacillus atrophaeus* genomic DNA extract were analysed in triplicates. The amount of DNA introduced in the assay ranged between 9 and 19,683 genome copies.

**Fig 6:**
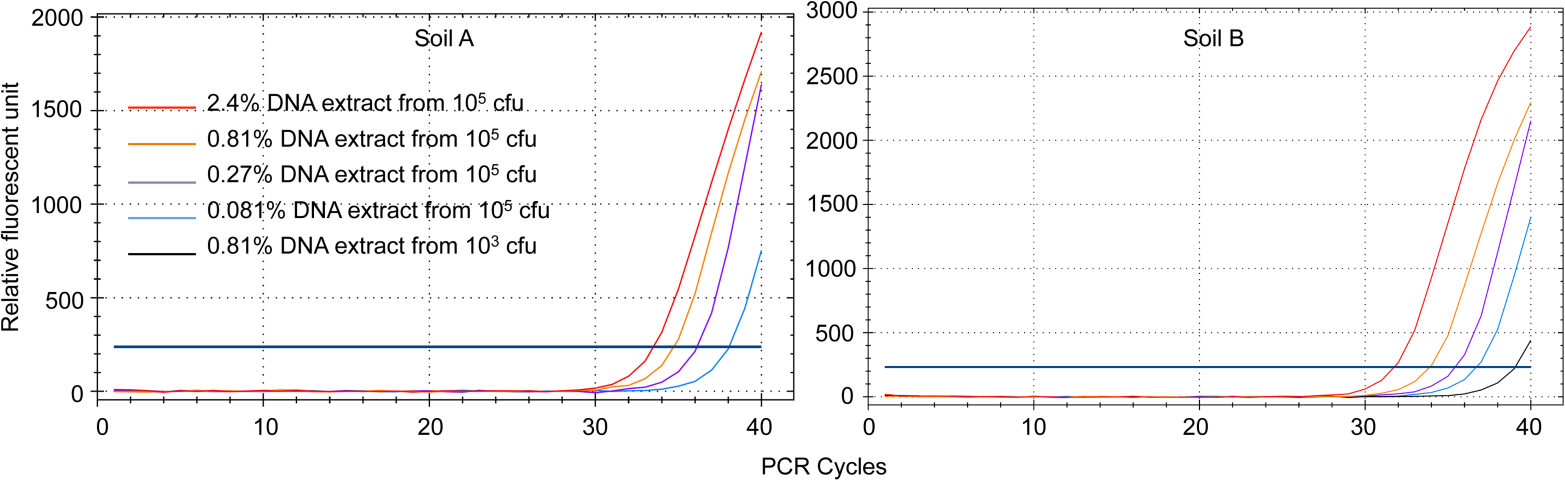
Real-time PCR analysis of *Bacillus atrophaeus* DNA in soil samples. Serial dilutions (from 2.4% to 0.081%) of the DNA extracts for control soil samples and soil samples inoculated with 10, 10^3^, or 10^5^ *Bacillus atrophaeus* cfu were analysed using a TaqMan-PCR assay. The figure shows representative amplification plots for PCR triplicates. Control soil samples and soil samples inoculated with 10 *Bacillus atrophaeus* cfu never yielded a PCR signal. The blue horizontal line corresponds to the threshold for the determination of the *C*_T_.

In order to compare the sensitivity of metagenomic sequencing and PCR to detect *Bacillus atrophaeus* DNA in soil samples, we performed real-time PCR studies. Fig 5 demonstrates the high sensitivity of the TaqMan real-time PCR assay used in this study. This assay displays optimal amplification efficiency (102%, as estimated from the slope of the plot). We next performed PCR on serial dilutions of the soil DNA extracts, including an amount of extract (0.81 μl, *i*.*e*. 0.81% of the DNA extract) comparable to that used to produce the metagenomic libraries. Fig 6 shows that, using this amount of DNA extract, *Bacillus atrophaeus* DNA was clearly evidenced in soil samples inoculated with 10^5^ cfu. It is worth mentioning that the PCR *C*_T_ was lower for soil B (34 cycles) than for soil A (34.7 cycles), which agrees with the observation that metagenomic sequencing yielded a higher number of DNA reads mapping to the *Bacillus atrophaeus* genome for the soil B than for the soil A extract (see Table 2 and Fig 4). For samples inoculated with 10^3^ cfu, amplification carried out using 0.81% of the soil A DNA extract never yielded a PCR signal. For soil B, the same amount of the DNA extract barely enabled DNA detection (2 PCR with a *C*_T_ of ~ 39 out of 8 replicates from 2 experiments). Finally, for control soils as well as soils inoculated with 10 *Bacillus atrophaeus* cfu, analysis of 0.81% of the extract never yielded a PCR signal.

## Discussion

The reference method to detect trace amounts of nucleic acids has long consisted in PCR-based single target amplification. With the advent of real-time PCR, the analytical process was sped up, and detection methods relying on hydrolysis probes (e.g. TaqMan-MGB probes [37]) or molecular beacons [38] further improved the specificity of the assays. A number of quantitative real-time PCR assays for biowarfare agents or simulants have been described [2, 39]. They usually allow detecting 10–100 DNA copies. For 1–10 copy numbers, PCR amplification is not recorded in all reaction tubes [11, 37], and detecting less than one genomic copy is impossible with any PCR assay. Isothermal DNA amplification has been considered as an alternative to quantitative PCR [40–41], but it is not amenable to detect less than one genomic copy.

The tremendous progress of next generation DNA sequencing technologies, whose throughput on a single machine increased by more than three orders of magnitude in the last ten years [42], opens a wealth of opportunity for these methods, including the survey of environmental samples. In the present study we focused on the Illumina method, which rests on the sequencing of libraries produced from DNA molecules sheared into relatively small fragments. Be et al. [19] already demonstrated that the Roche 454 and Illumina GaIIx platforms are much more efficient than hybridization arrays to detect *Bacillus anthracis* DNA spiked into DNA retrieved from aerosol or soil samples. The sensitivity achieved in their study, using whole genome amplification before generating the libraries, was in the range of 10–100 GEs, depending on the sample (aerosol or soil DNA extract) and the sequencing technology. Here we used crude environmental samples by coextracting the DNA from endogenous organisms and inoculated *Bacillus atrophaeus* cells. To speed up the processing of the samples, we omitted the whole genome amplification step. Based on the observation that large amounts of the soil DNA extracts decrease the yield of DNA produced by PCR, we used amounts corresponding to 0.83% of the extracts to produce the PCR-amplified metagenomic libraries (see Materials and Methods).

In soil samples inoculated with 10^5^ cfu, we recorded 939 reads for a total number of 125,261,786 reads (7.5 ppm) for soil A, and 4,633 reads for a total number of 148,385,427 reads (31 ppm) for soil B aligning to the *Bacillus atrophaeus* genome but to none of the other *Bacillus* sp. genomes (Table 2). The sensitivity achieved for soil A is therefore less than 10 ppm of the total number of reads.

Regarding the samples inoculated with 10^3^ cfu, in soil A the relative increase of the number of reads aligning to the *Bacillus atrophaeus* genome, as compared to the non-inoculated sample, was in the range of the variations recorded for other *Bacillus* sp. A more dramatic increase was observed in soil B. Interestingly, PCR analysis of the soils inoculated with 10^3^ cfu either failed to evidence *Bacillus atrophaeus* DNA (soil A) or yielded an amplification signal in a minority of replicate samples (soil B). This suggests that the sensitivity of our metagenomic strategy is in the range of that of the PCR assay.

It has to be noticed that if we try to estimate the number of *Bacillus atropheus* genome copies in our total DNA extract from the results of the PCR analysis, we get very low amounts. Regarding for example the soil B sample inoculated with 10^5^ *Bacillus atropheus* cfu, the *C*_T_ of 34 cycles obtained for 0.81% of the DNA extract (Fig 6) corresponds to about 35 *Bacillus atrophaeus* genome copies (Fig 5). According to these calculations, the 100-μl DNA extract contains 4,000 *Bacillus atrophaeus* genome copies. Even if we consider the minimum number of one *Bacillus atrophaeus* genome copy per cfu, the yield of the DNA extraction process in this case is only of 4%. A similar low extraction yield has been reported for a variety of bacterial cells using this method [43]. A mixture of beads of different diameters was shown to be efficient to increase the DNA yield from a variety of cells, including gram-positive bacterial vegetative cells and spores [24]. Thus, a much better DNA yield should be obtained by replacing the single-size garnet beads of the extraction beads by a mixture of different beads. Additional modifications to the extraction process could consist in performing the cell-lysing step in sodium phosphate buffer, a procedure that was successfully used to extract DNA from clay-rich sediments [44–45]. The DNA extraction step is indeed a critical one, and improving the DNA extraction yield should lead to an increased sensitivity for both PCR and metagenomic analyses.

**Fig 7:**
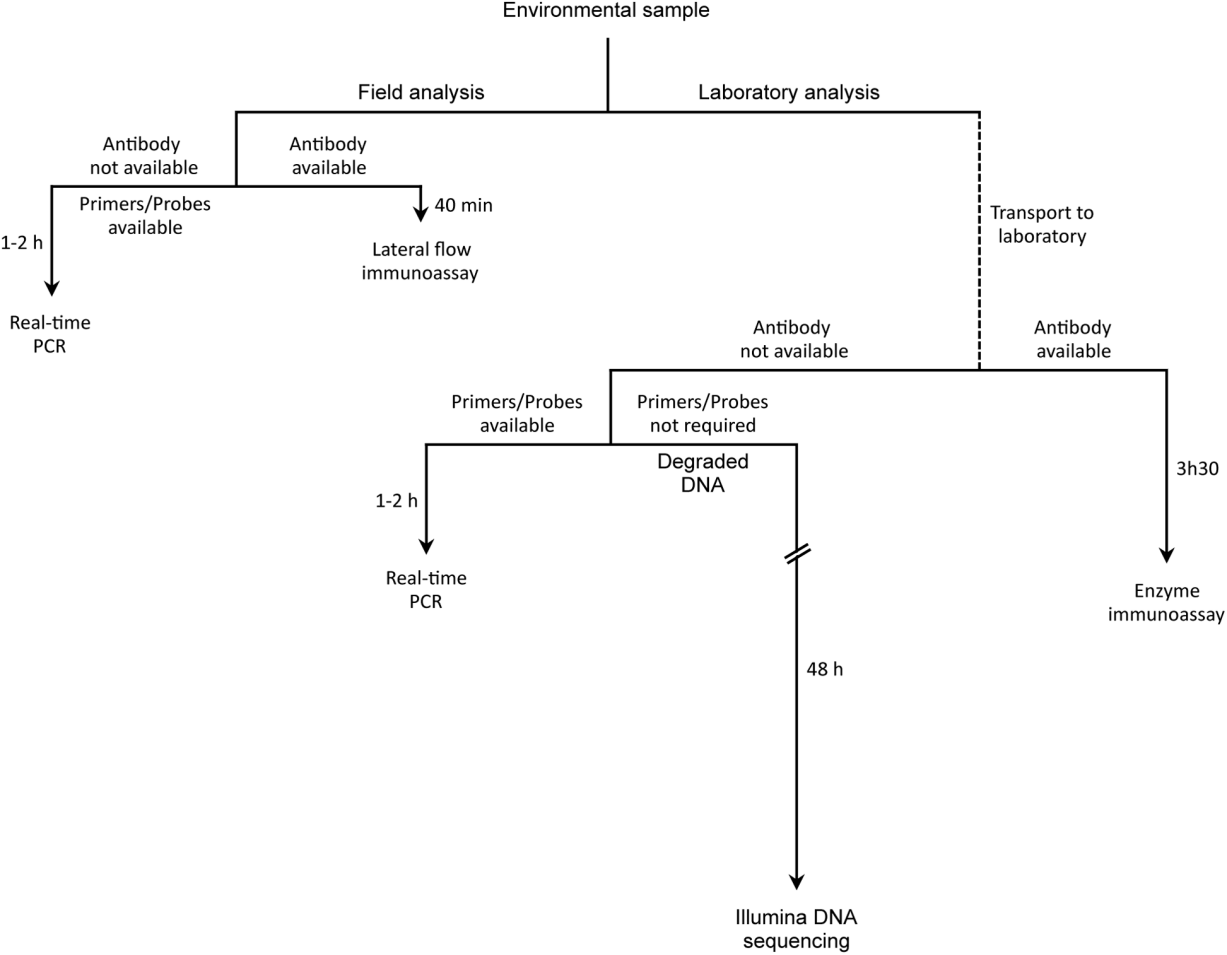
Guidelines for selecting a method to detect DNA or protein from a target organism in an environmental sample.

A comparison of the sensitivity, and guidelines for selecting methods to analyse environmental samples for DNA or protein content are provided in S2 Table and in Fig 7. In S2 Table, for comparative purposes values from the literature [5–6, 8, 11, 12, 19, 46–48] are presented as absolute amounts (*i*.*e*. cfu or GEs) rather than concentration (cfu/ml, GEs/ml) in the assayed sample. Whatever the method, a matrix effect is observed so that the sensitivity achieved tends to decrease with the complexity of the sample [5, 19, 46–47]. Concerning detection with antibodies, enzyme immunoassays carried out in microplates are much more sensitive than lateral flow (*i*.*e*. dipstick) immunoassays [5]. When standard TaqMan PCR assays were compared to TaqMan array card assays, a much better sensitivity was reported for the former procedure than for the later [11]. The 454 and Illumina DNA sequencing platforms display comparable sensitivities that are much better than those using hybridization on microarrays [19].

With these considerations in mind, identifying the most relevant assay for analysing environmental samples will first depend on the facilities available (Fig 7). When the analysis has to be carried out on the field, the fastest way (~40 min) to obtain the results would be to perform a lateral flow immunoassay. However, when antibodies are not available or when high sensitivity is required, a PCR assay using a portable device would be used. This approach can deliver results in 1h or less when a crude sample is used, and within 2h if DNA extraction has to be performed. When the analysis is carried out in the laboratory and antibodies are available, enzyme immunoassays would be preferred to lateral flow immunoassays because of much higher sensitivity. In the absence of antibodies, real-time PCR is a relevant choice when the target is characterized well enough to design primers. Examples for this include the analysis of virulence genes. To guarantee specific detection, a TaqMan assay would be preferred to the SYBR green detection method. The shotgun DNA sequencing method used here and in previous studies displays a sensitivity comparable to that of PCR but it is obviously much more labour intensive. However, in the framework of bioterrorism, detection methods need to be implemented immediately, sometimes even before the full characterization of the agent is complete. The method described is equally sensitive as PCR, but it does not require primer development, which can be extensive, particularly in taxonomic ranges where reference sequences are uncommon. With the presented method, the genomic characterization of the agent could occur as part of the detection process. As the price and sensitivity of next generation sequencing improve, this advantage may be the difference between effective and ineffective responses to an attack. In addition, whereas real-time PCR is only useful for DNA fragments longer than 60 bp, shotgun sequencing can successfully analyse highly degraded DNA consisting of 30–40 bp fragments, as shown by ancient DNA studies carried out by us and other laboratories [25, 49].

In the present study, DNA sequencing was carried out using the normal mode of the Illumina Hiseq 2500 platform, which required 5 days. The sequencing process could be reduced to 1 day by using the rapid mode on the same platform or more recent platforms with even higher throughput. Concerning data analysis, our bioinformatic processing of DNA reads (including the alignment to a set of bacterial genomes) can be performed within 2 hours on a personal computer equipped with a 2.3 GHz processor. The total time for a metagenomic analysis could thus be reduced from about 6 days (present study) to two days (Fig 7). Accordingly, although the current cost of high-throughput sequencing is much higher than that of a PCR assay, the benefit of the approach warrants consideration. In addition, metagenomic sequencing makes it possible to reconstruct complete genome sequences [50], which is of interest to uncover biothreat organisms whose genome portions have been modified to escape detection using standard tests. However it should be stressed that in this case the coverage obtained by DNA sequencing should be high enough to provide information on all genome portions. Finally, metagenomics should be especially useful to search for highly degraded DNA in laboratories that were bleached to mask the illicit manipulation of biowarfare agents.

## Supporting information

S1 Table Number of reads mapping to the indicated *Bacillus* sp. genomes. Columns 2–5 and 6–9 indicate the number of reads mapped to each genome independently (see Methods) for each soil sample. Soil samples were inoculated with 0 to 10^5^ *Bacillus atrophaeus* cfu, and each library was produced from 0.83% of the total DNA extract.
(DOCX)

S2 Table Comparison of the sensitivity of different methods for analysing a variety of virus and bacterial species. ^a^Values correspond to the absolute amount of material introduced in the assay. EIA: enzyme immunoassay; MS: mass spectrometry; cfu: colony forming unit; GEs: genomic equivalents.
(XLSX)
