# Supplementary material for: Comparative analysis of the sensitivity of metagenomic sequencing and PCR to detect a biowarfare simulant (Bacillus atrophaeus) in soil samples
Source: PLoS One. 2017 May 4;12(5):e0177112. doi: 10.1371/journal.pone.0177112 (PMC5417559; doi:10.1371/journal.pone.0177112)
Supplement: S1 Table — Columns 2–5 and 6–9 indicate the number of reads mapped to each genome independently (see Methods) for each soil sample. Soil samples were inoculated with 0 to 105 Bacillus atrophaeus cfu, and each library was produced from 0.83% of the total DNA extract. (DOCX) [file pone.0177112.s001.docx]

**S1 Table. Number of reads mapping to the indicated *Bacillus* sp*.* genomes.**

| Species | Soil A / 10^8^ reads | | | | Soil B / 10^8^ reads | | | |
| --- | --- | --- | --- | --- | --- | --- | --- | --- |
|  | 0 | 10 | 10^3^ | 10^5^ | 0 | 10 | 10^3^ | 10^5^ |
| *Bacillus atrophaeus* | 2 | 2 | 11 | 754 | 0 | 1 | 40 | 3128 |
| *Bacillus amyloliquefaciens* | 0 | 0 | 2 | 1 | 1 | 0 | 0 | 1 |
| *Bacillus anthracis str. 'Ames Ancestor'* | 9 | 16 | 4 | 4 | 3 | 0 | 10 | 5 |
| *Bacillus anthracis str. Sterne* | 9 | 16 | 4 | 4 | 3 | 0 | 10 | 5 |
| *Bacillus cereus* | 7 | 15 | 5 | 4 | 6 | 1 | 12 | 5 |
| *Bacillus licheniformis* | 17 | 16 | 30 | 17 | 0 | 0 | 2 | 1 |
| *Bacillus megaterium* | 69 | 83 | 63 | 65 | 3 | 2 | 2 | 0 |
| *Bacillus mycoides* | 55 | 106 | 33 | 76 | 20 | 13 | 127 | 18 |
| *Bacillus pseudomycoides* | 75 | 161 | 165 | 167 | 89 | 112 | 226 | 168 |
| *Bacillus pumilus* | 3 | 3 | 5 | 3 | 1 | 1 | 2 | 0 |
| *Bacillus subtilis* | 3 | 5 | 8 | 6 | 0 | 0 | 0 | 5 |
| *Bacillus thuringiensis* | 7 | 13 | 4 | 6 | 3 | 1 | 14 | 4 |
| *Bacillus weihenstephanensis* | 89 | 162 | 118 | 145 | 53 | 52 | 153 | 93 |
| *Geobacillus stearothermophilus* | 0 | 3 | 2 | 1 | 0 | 0 | 0 | 0 |
| *Lysinibacillus sphaericus* | 0 | 1 | 0 | 0 | 1 | 2 | 0 | 1 |

Columns 2-5 and 6-9 indicate the number of reads mapped to each genome independently (see Methods) for each soil sample. Soil samples were inoculated with 0 to 10^5^ *Bacillus atrophaeus* cfu, and each library was produced from 0.83% of the total DNA extract.
